# Metabolomic Profiling of *Tenebrio molitor* Reared on Chestnut Shell-Enriched Substrate Using NMR Spectroscopy

**DOI:** 10.3390/foods13233757

**Published:** 2024-11-24

**Authors:** Irene Ferri, Mattia Spano, Matteo Dell’Anno, Luisa Mannina, Luciana Rossi

**Affiliations:** 1 Department of Veterinary Medicine and Animal Sciences—DIVAS, University of Milan, Via dell’Università 6, 26900 Lodi, Italy; irene.ferri@unimi.it (I.F.); matteo.dellanno@unimi.it (M.D.); luciana.rossi@unimi.it (L.R.); 2 Food Chemistry Lab, Department of Chemistry and Technology of Drugs, Sapienza University of Rome, 00185 Rome, Italy; luisa.mannina@uniroma1.it

**Keywords:** mealworm larvae, *Castanea sativa*, growth substrate, metabolomics, by-products, insect meals, Nuclear Magnetic Resonance spectroscopy, fumarate, uracil, fatty acids

## Abstract

The aim of this study was to evaluate the metabolomic profile of *T. molitor* larvae reared on the following innovative growth substrates: wheat bran (control, CTRL); wheat bran supplemented with 12.5% *w*/*w* chestnut shell (TRT1); and wheat bran supplemented with 25% *w*/*w* chestnut shell (TRT2) for 14 days of trial. At the end of this experiment, larvae were transformed into insect meals for nutritional characterization. Nuclear Magnetic Resonance (NMR) spectroscopy was carried out to evaluate the metabolomic profile of organic acids, sugars, nitrogen bases and derivates, fatty acids, and other compounds. Chemical analysis showed an increased level of crude protein in TRT1 compared to CTRL and TRT2 (*p =* 0.0391). The metabolite profiles of TRT1 and TRT2 were similar to each other but distinct from those of the CTRL group. Notably, larvae enriched with chestnut shells revealed the presence of uracil, uridine, and glucose, while fumarate was absent. The enrichment analysis showed that in TRT1 and TRT2, the glyoxylate and dicarboxylate metabolism was more relevant compared to CTRL. These findings indicate that chestnut shell inclusion affects the larvae metabolism of *T. molitor* and demonstrates the effectiveness of NMR spectroscopy in revealing a relation between insect metabolism and growth substrate.

## 1. Introduction

The request for alternative protein sources in animal nutrition has become imperative, particularly to reduce the European protein gap. In the European Union, the dependency on soybean imports, the primary protein used in animal feed, poses significant economic and environmental challenges [1]. In this scenario, insects have emerged as one of the most promising alternative protein sources [2]. Among these, *Tenebrio molitor* (yellow mealworm) is gaining interest as a feed ingredient for monogastric animals, offering high concentrations of essential nutrients with potential additional functional properties such as antioxidant and antimicrobial activities [3].

The nutritional profile of *Tenebrio molitor* is remarkable, with protein content ranging from 40% to 70% of its dry matter and varying during the life cycle stage and rearing conditions [4]. The quality of insect protein is not only considered in terms of the total amount but also in terms of the high biological value of the amino acid profile. The sustainability of yellow mealworms is further enhanced by their ability to be reared on agro-industrial by-products, thereby valorizing waste and potentially increasing the nutritional profile of *T. molitor* by providing bioactive compound-rich sources [5,6]. The ability of bioconversion performed by insects not only contributes to waste reduction but also aligns with circular economy principles, enhancing the environmental value of insect farming [7,8].

The utilization of insects in animal feed could revolutionize livestock farming by integrating insect rearing into existing farming systems, thus creating local insect-based protein production. Yellow mealworms can be raised on agro-industrial by-products that are, otherwise, unsuitable for feed purposes, thereby converting low-value plant materials into high-value protein products. This not only enhances the sustainability of protein production but also contributes to waste valorization and resource efficiency.

The evidence suggests that the growth substrate influences the nutritional profile of the resulting insect meal [9]. Various substrates have been explored, but wheat bran remains the predominant rearing substrate in insect farming. However, this by-product is in direct competition with animal feed formulation. Additionally, there is a lack of information regarding insect metabolism, which is involved in the key mechanisms of bioconversion and the production of functional compounds (e.g., antimicrobial peptides) with potential beneficial impacts on health. In this context, metabolomics is a powerful tool for analyzing metabolite output in a biological system, providing insights into the molecular response to various stimuli, such as dietary treatments [10]. In this regard, Nuclear Magnetic Resonance (NMR) spectroscopy is an advanced methodology that allows us to identify, within the same experiment and without any separation step, metabolites belonging to different chemical classes [11]. The potential advantages related to the NMR-based metabolomics approach can, thus, represent a potential tool to further define and clarify the chemical profile of innovative matrices, namely, edible insects. Up to now, this approach has only been applied to the analysis of spray-dried powder of *A. domesticus* [12] and the free amino acid profile of *T. molitor* larvae reared with wheat bran and brewer’s spent grains [13]. Within these studies, NMR metabolomics allowed us to identify, for the first time, some metabolites never identified in edible insects and metabolite profile modifications related to different growth substrates used in *T. molitor* larvae.

The current study relies on previous encouraging results, which showed that the supplementation of chestnut shells in the growth substrate enhanced the survival of *T. molitor* larvae, modified the amino acid composition, and improved their antimicrobial properties [14]. In light of this, the aim of this study was to evaluate the metabolites’ profile of *T. molitor* larvae reared on chestnut shell-supplemented growth substrate using NMR spectroscopy. By investigating these metabolic changes, this study aims to elucidate the potential benefits and drawbacks of incorporating chestnut shells into the diet of mealworms and to enrich the scientific knowledge on insect metabolism.

## 2. Materials and Methods

### 2.1. Rearing Conditions of Insects and Growth Substrates

A stock colony of *Tenebrio molitor* larvae from Italian Cricket farm s.r.l, a local farm in Pinerolo (Italy), was used for this study. Briefly, the larvae were reared on a traditional growth substrate of wheat bran and hydrated with vegetables until the beginning of the trial. At seven weeks, larvae were randomly allocated in 24 plastic trays (27 × 39 × 14 cm), eight replicates for treatment, for a total of 2.4 kg, corresponding to approximately 500 larvae per tray (100 g/tray). The experimental groups of larvae differed only for the growth substrate: the control group (CTRL, *n* = 8) was grown on 50g of wheat bran; treatment group 1 (TRT1) was reared on 50 g of wheat bran supplemented with 12.5% *w*/*w* chestnut shell; and treatment group 2 (TRT2) received 50 g of wheat bran supplemented with 25% *w*/*w* chestnut shell, Table 1. The substrates were substituted weekly to administer feed ad libitum for 14 days of the trials [14]. The composition of different substrates is illustrated in Table 1. Chestnut shells, consisting of the pericarp and integument of the fruit, have been recovered as wastes of the chain production of the Luciniera Farm (Modena, Italy). It was dried and ground before being used in a growth substrate. At days 0, 2, 4, 6, 8, 10, and 12, larvae received 10 mL of water as a hydration source. Water was sprayed directly on the substrate [8]. During this experiment, insects were maintained at 26 ± 2 °C and 60–75% relative humidity, corresponding to the ideal conditions for rearing *T. molitor* larvae.

### 2.2. Chemical Characterization of Growth Substrates

In accordance with the “Official Methods of Analysis” [15], the chemical composition of growth substrates was assessed. Samples were placed in pre-weighed aluminum bags and dried in a forced-air oven set at 65 °C for 24 h in order to determine the dry matter (DM) (AOAC method 930.15). Ethyl ether was used in a Soxtec extractor (AOAC 2003.05) to measure the lipid content (ether extract, EE). The total ash content was determined after three hours of incineration at 550 °C (AOAC method 942.05). A Kjeldahl methodology was used to calculate crude proteins (CP) by applying a nitrogen conversion factor of 6.25 for growth substrates (AOAC method 2001.11). The crude fiber content of the growth substrates was determined using the AOACS Ba 6a-05 method through filtering bags. Finally, non-structural carbohydrates of growth substrates were calculated through the following equation:

NSCs = 100 − (moisture% + ash% + EE% + CP% + CF%)(1)

Growth substrates were analyzed in technical triplicate, and the analysis procedure was repeated three times for each group.

### 2.3. Evaluation of Total Polyphenol Content (TPC) of Growth Substrates

Total polyphenol content was determined according to Attard [16], with minor modifications. Briefly, 2.5 g of each sample was diluted in 15 mL of methanol and stirred for 48 h at room temperature. After centrifugation (5000 rpm, 10 min), the supernatants were collected and used for further determination of total polyphenol content (TPC).

TPC was analyzed through the Folin–Ciocalteau reagent with a microtiter plate assay, using tannic acid as the standard (960, 480, 240, 120, 60, 30, 15, 7.5, and 0 ug/mL). The reaction mixture consisted of 10 uL of each sample, 100 uL of Folin–Ciocalteau reagent (diluted 1:10, *v*/*v* with deionized water), and 80 uL of sodium carbonate solution (1 M). After 20 min of incubation at room temperature, absorbances were measured at 630 nm using a microplate reader (BioTek Synergy HTX Multimode Reader, Agilent Technologies, Santa Clara, CA, USA) [17]. The results were expressed as mg of tannic acid equivalents/100 g of sample (mg TA Eq/100 g).

### 2.4. Chemical Characterization of Tenebrio Molitor Larvae Meal

After 14 days from the beginning of the trial, when the first pupae began to appear, all the larvae for tray were harvested, separated from the growth substrate with a mesh sieve (ø 300 µm), and weighed. Larvae were starved for 24 h before being transformed into insect meals to ensure that the contents of the digestive tract of the larvae did not impact the results of the nutrient composition analysis of the larvae. After that, insects were killed by freezing at −20 °C, and each tray of insects (8 replicates/group) was cooked through a microwave (model CMG2071M, Candy Hoover Group S.r.l., Brugherio, Italy) at 120 W with a frequency of 2450 MHz for 5 min, followed by grinding to obtain insect meals. The chemical composition of *T. molitor* meals was evaluated separately as independent replicates for each group (*n* = 8) through the “Official Methods of Analysis”, as previously described.

### 2.5. NMR Analysis and Enrichment Analysis

For NMR metabolomics analysis, the Bligh–Dyer extract procedure was applied since the use of solvents with different polarities allowed us to extract both polar and apolar metabolites. In particular, 100 mg of sample were added with 3 mL of CH_3_OH/CHCl_3_ 2:1 *v*/*v* mixture and 0.8 mL of distilled water. After sonication, 1 mL of CHCl_3_ and 1 mL of H_2_O were added, and the two-phase system was centrifugated, allowing for the separation of hydroalcoholic and organic phases. The extraction procedure was applied two more times to the residual pellet, and both hydroalcoholic and organic phases were dried under N_2_ flux.

For NMR analysis, hydroalcoholic dried extracts were solubilized in 1 mL of 100 mM phosphate buffer/D_2_O, containing 0.5 mM TSP (3-(trimethylsilyl)propionic acid sodium salt) as internal standard and, after centrifugation to remove any solid residual, 700 µL of this solution were transferred into a 5 mm NMR tube. Organic dried extracts were solubilized in 1 mL of CD_3_Cl/MeOD 2:1 *v*/*v* mixture, and after centrifugation to remove any solid residual, 700 µL of this solution was transferred into a 5 mm NMR tube.

Analyses were carried out with a 600 MHz spectrometer (Jeol JNM-ECZ 600 R) equipped with a 5 mm probe FG/RO DIGITAL AUTOTUNE. ^1^H NMR experiments were conducted using the same acquisition and processing parameters previously reported for the analysis of edible insects in the same conditions [12].

For hydroalcoholic extract metabolites, data were obtained referring to TSP and expressed as mg/100 g sample ± SD (*n* = 3). For organic extract metabolites, data were expressed as molar % ± SD (*n* = 3) as a result of the following equations based on relative areas of comparison:

%STE = 100(0.66*I_STE_/I_tot_) (2)

%TUFA = 100*(0.5*I_TUFA_/I_tot_) (3)

%DUFA = 100*(I_DUFA_/I_tot_) (4)

%TOT UFA = 100*(0.5*I_TOT UFA_/I_tot_) (5)

%MUFA = %TOT UFA − %DUFA − %TUFA(6)

%TOT FA = 100*(I_TOT FA_/I_tot_) (7)

%TOT SFA = %TOT FA − %TOT UFA(8)

with %STE, %TUFA, %DUFA, %MUFA, %TOT FA, %TOT UFA, and %TOT SFA being the molar % of sterols, tri-unsaturated fatty acids, di-unsaturated fatty acids, mono-unsaturated fatty acids, total fatty acids, total unsaturated fatty acids, and total saturated fatty acids, respectively.

I_STE_, I_TUFA,_ I_DUFA_, I_TOT UFA_, and I_TOT FA_ are the integral areas of sterols, tri-unsaturated fatty acids, di-unsaturated fatty acids, total unsaturated fatty acids, and total fatty acids signals, respectively. I_tot_ is obtained from the equation below:

I_tot_ = I_TOT FA_ + 0.66*I_STE_ (9)

Enrichment analysis was carried out on the obtained NMR metabolomics data to identify the metabolic pathways of both *Tenebrio molitor* control and treatment groups, including the information on amino acid profiles from analysis in the previous study [14]. MetaboAnalyst 6.0 software was used for this purpose, referring to KEGG pathway databases [18].

### 2.6. Statistical Analysis

All data were analyzed using GraphPad Prism statistical software (Version 9.1.1). In particular, a one-way analysis of variance (ANOVA) was performed after a statistical test for normality (Shapiro–Wilk test) and homoscedasticity (Bartlett’s test). Post-hoc Tukey’s test was used to separate means during the multiple comparisons. Student’s *t*-test was used to analyze values measured only in two groups. The multivariate analysis of the principal component analysis (PCA) was performed to analyze data on the metabolite profiles of different larvae groups and detect clustering patterns. Values were presented as means ± standard error, and differences were considered statistically significant for *p* < 0.05.

## 3. Results

### 3.1. Nutrient Composition of Growth Substrates

The chemical composition of the experimental growth substrates is shown in Table 2. The protein levels were significantly different between the groups. CTRL substrate registered higher protein levels compared to TRT1 and TRT2 groups (*p* < 0.05).

### 3.2. Total Polyphenols Content in Growth Substrate

The results showed that the TRT2 growth substrate had the highest total polyphenol content, measuring 770.80 ± 32.60 mg TA Eq/100 g (*p* < 0.0001). The TPC in the TRT1 substrate reached 220.61 ± 27.41 mg TA Eq/100 g, while the CTRL group substrate had the lowest TPC, with 152.01 ± 24.71 mg TA Eq/100 g (*p* < 0.0001).

### 3.3. Tenebrio Molitor Meal Chemical Characteristics

At the end of this trial, comparable larvae biomass was obtained from each group, with values equal to 104.9 g, 109.4, and 112.3 g for CTRL, TRT1, and TRT2, respectively. DM values of CTRL (93.3 ± 3.35) were higher than those of TRT1(81.45 ± 7.64) and TRT2 (82.87 ± 2.24) (*p* = 0.0002). TRT1 registered the highest content of CP (51.96 ± 6.89) compared to the CTRL (44.52 ± 2.96) and TRT2 (46.22 ± 6.23) (*p* = 0.0391). Lipids in TRT1 (36.09 ± 4.40) were slightly higher compared to those in TRT2 (32.51 ± 1.21) and significantly different from the CTRL (31.14 ± 2.79) (*p* = 0.0123).

### 3.4. Metabolite Profile of Tenebrio Molitor Meals

NMR analysis of both hydroalcoholic and organic Bligh–Dyer extracts allowed us to identify metabolites belonging to several chemical classes, namely, organic acids, sugars, nitrogen bases and derivatives, fatty acids, sterols, and other compounds. The metabolites identification was obtained by combining the information contained in ^1^H spectra (chemical shifts value, signal multiplicity, *J*-coupling constants) and the literature data regarding edible insects analyzed in the same or similar experimental conditions [12,13].

The identified metabolites, together with ^1^H NMR signals (chemical shift, multiplicity, *J*-coupling constant) selected for quantification, are reported in Table 3. Due to structural similarity and signal overlapping, sterols, total unsaturated fatty acids, fatty acids, and total fatty acids were identified as the main chemical groups; thus, signal ranges were used for their quantification.

**Figure 1:**
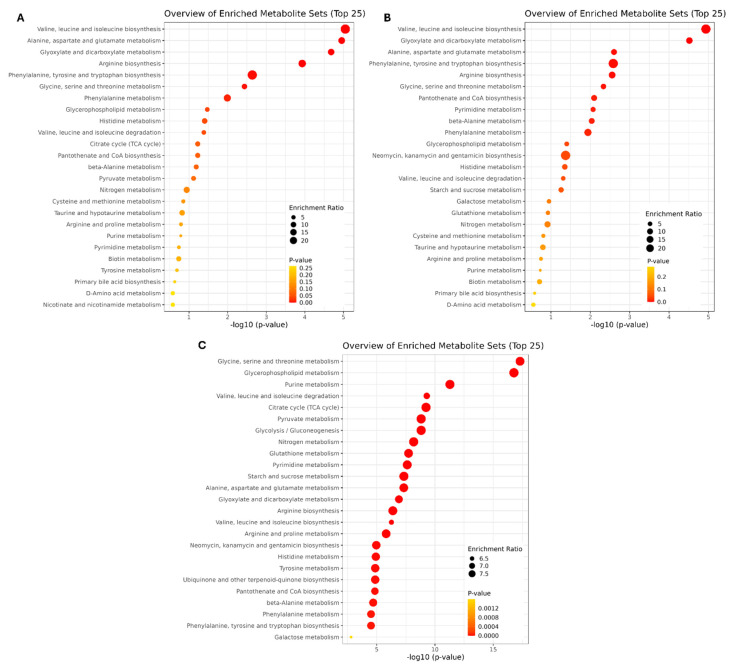
Metabolic pathway analysis of *T. molitor* samples. Over-Representation Analysis of CTRL (**A**) and TRT (**B**) metabolic pathways. Quantitative Enrichment Analysis to compare CTRL and TRT groups (**C**).

Enrichment analysis was also carried out using data relative to amino acids previously reported [14]. In particular, Over-Representation Analysis was carried out on CTRL and TRT groups to define the metabolic pathways mainly involved in the two groups (Figure 1A,B). Both the CTRL and treatment groups showed valine, leucine, and isoleucine synthesis as the main metabolic pathway. A similar metabolic pathway profile was observed in the CTRL group compared to the chestnut peel-supplemented treatments. However, the treatment groups exhibited a higher probability of promotion of glyoxylate and dicarboxylate metabolism, whereas the CTRL group predominantly showed activation of alanine, aspartate, and glutamate metabolism. Quantitative Enrichment Analysis was applied to compare the quantitative variations between CTRL and TRT groups (Figure 1C).

### 3.5. Organic Acids

**Figure 2:**
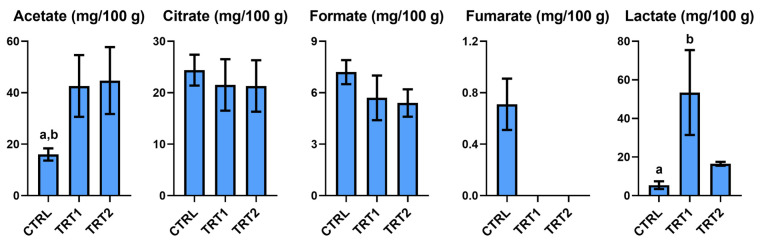
Organic acids quantified in Bligh–Dyer hydroalcoholic extracts of *Tenebrio molitor* meals of different growth substrates: wheat bran (CTRL) and wheat bran with 12.5 and 25% chestnut shell (TRT1 and TRT2). Values are expressed as mg/100 g of sample dry weight ± SD (*n* = 3). One-way ANOVA, followed by Tukey’s test, was applied to underline significant differences (*p* < 0.05): (a) vs. TRT1; (b) vs. TRT2.

The analysis of the effect on organic acids of *T. molitor* larvae revealed significant differences among the groups, except for citrate (Figure 2). In general, all replicates for treatment presented a variable trend for the detected organic acids. Specifically, acetate and formate were comparable in TRT1 and TRT2 and differed from the CTRL group, which showed lower content of acetate. In contrast, the formate concentration was higher in the larvae of the CTRL group than in the treatment groups. The highest level of lactate was registered in TRT1.

### 3.6. Sugars

**Figure 3:**
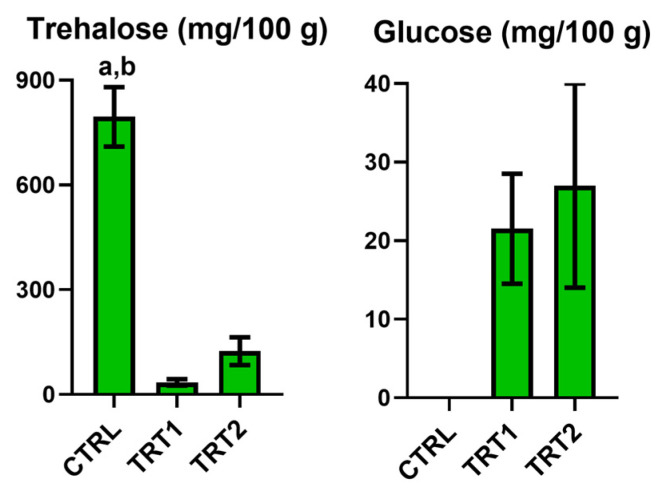
Sugars quantified in Bligh–Dyer hydroalcoholic extracts of *Tenebrio molitor* meals of different growth substrates: wheat bran (CTRL) and wheat bran with 12.5 and 25% chestnut shell (TRT1 and TRT2). Values are expressed as mg/100 g of sample dry weight ± SD (*n* = 3). One-way ANOVA, followed by Tukey’s test, was applied to underline significant differences (*p* < 0.05): (a) vs. TRT1; (b) vs. TRT2.

Trehalose was variable within each replicate for the group; however, the CTRL showed higher concentration with significant differences than the TRT1 (*p* = 0.0002). In contrast, glucose was absent in the CTRL, with similar levels between TRT1 and TRT2 (Figure 3).

### 3.7. Nitrogen Bases and Derivatives

**Figure 4:**
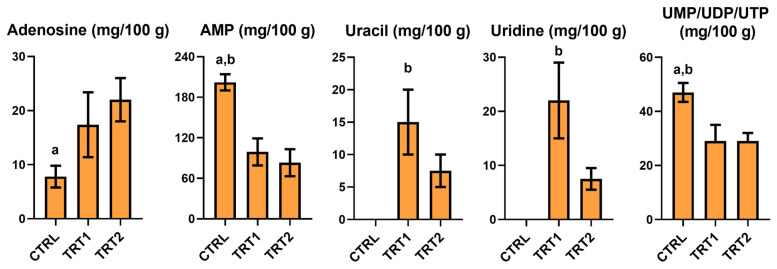
Nitrogen bases and derivatives quantified in Bligh–Dyer hydroalcoholic extracts of *Tenebrio molitor* meals of different growth substrates: wheat bran (CTRL) and wheat bran with 12.5 and 25% chestnut shell (TRT1 and TRT2). Values are expressed as mg/100 g of sample dry weight ± SD (*n* = 3). One-way ANOVA followed by Tukey’s test was applied to evaluate significant differences (*p* < 0.05): (a) vs. TRT1; (b) vs. TRT2.

The qualitative and quantitative profiles of nitrogenous bases and their derivatives exhibited variability in each sample analyzed across all groups (8 replicates per group) (Figure 4). AMP was higher in CTRL compared to TRT groups. A similar trend was reported for UMP/UDP/UTP, observing higher values in the CTRL group compared to TRT1 and TRT2. Uracil was detected only in the insect reared on chestnut shell with lower values on TRT1 than TRT2. TRT2 showed the numerically higher value of adenosine with significant differences from the CTRL (*p* = 0.0058). TRT1 and TRT2 highlighted levels of uridine, which was absent in the CTRL group.

### 3.8. Fatty Acids

**Figure 5:**
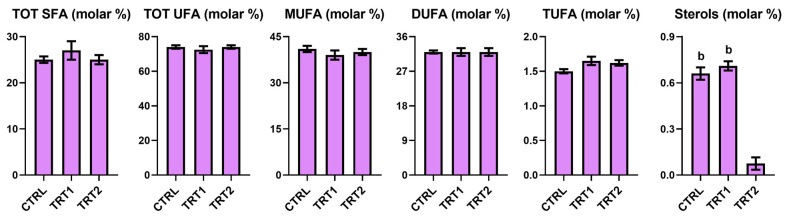
Fatty acids and sterols quantified in Bligh–Dyer organic extracts of *Tenebrio molitor* meals of different growth substrates: wheat bran (CTRL) and wheat bran with 12.5 and 25% chestnut shell (TRT1 and TRT2). Values are expressed as molar percentage ± SD (*n* = 3). One-way ANOVA, followed by Tukey’s test, was applied to underline significant differences (*p* < 0.05): (b) vs. TRT2.

The relative content of lipidic fraction exhibited no variation across the samples from different groups (Figure 5), underlying that chestnut shell implementation did not affect the lipid metabolism of *T. molitor*. The only significative difference was observed for sterols in the TRT2 group, with a four-time decrease in the relative content.

### 3.9. Other Compounds

**Figure 6:**
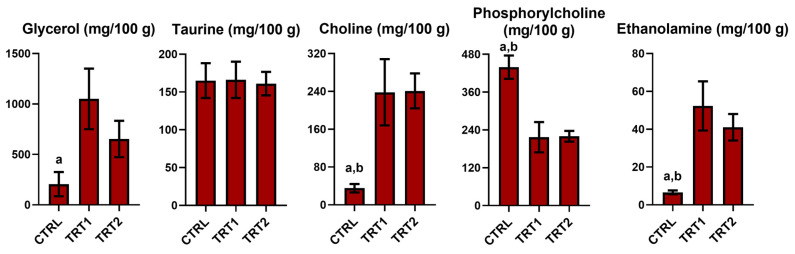
Other compounds quantified in Bligh–Dyer hydroalcoholic extracts of *Tenebrio molitor* meals of different growth substrates: wheat bran (CTRL) and wheat bran with 12.5 and 25% chestnut shell (TRT1 and TRT2). Values are expressed as mg/100 g of sample dry weight ± SD (*n* = 3). One-way ANOVA, followed by Tukey’s test, was applied to underline significant differences (*p* < 0.05): (a) vs. TRT1; (b) vs. TRT2.

Regarding other compounds detected in *T. molitor*, ethanolamine, choline, and glycerol increase in TRT1 and TRT2 with significant differences compared to CTRL, Figure 6. Moreover, TRT1 was particularly rich in glycerol, with values significantly different from the other groups.

### 3.10. Multivariate Analysis

**Figure 7:**
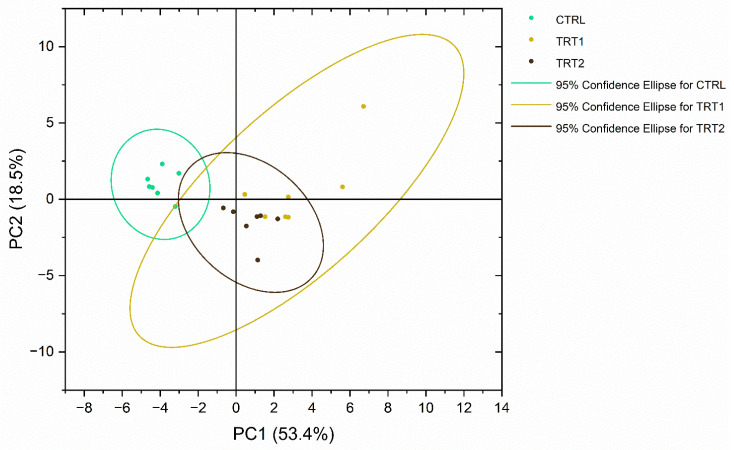
Principal component analysis (PCA) of ^1^H NMR of metabolites profiles acquired on CTRL (wheat bran), TRT1 (wheat bran and 12.5% of chestnut shell), and TRT2 (wheat bran and 25% of chestnut shell) larvae groups.

The metabolite profiles of sample meals highlighted distinct clustering in the PCA plot for larvae that received chestnut shell supplementation in the growth substrate compared to the CTRL group, Figure 7. Furthermore, samples of TRT2 displayed a more homogeneous distribution compared to TRT1, which showed higher variability.

## 4. Discussion

In the current study, NMR spectroscopy was employed to analyze the metabolomic profile of *T. molitor* larvae and assess a potential correlation between substrate composition and the metabolic response in these insects. To investigate the mechanism underlying the larvae’s nutrient utilization efficiency in relation to the intake of different ingredients, a preliminary chemical characterization of the substrates was performed. This analysis revealed differences in protein content, with the CTRL group exhibiting the highest levels. The evaluation of total polyphenol content in the growth substrates revealed a progressive increase in polyphenol levels in line with the rising inclusion of chestnut shells. Consistent with the survival rates observed in the previous study, polyphenol concentrations were significantly higher in the TRT2 treatment group compared to TRT1 and CTRL. This suggests that the higher polyphenol levels, likely due to their antioxidant properties, may have contributed to the enhanced survival of the insects in the previous study [14].

NMR metabolic profiling further revealed no presence of fumarate in TRT1 and TRT2 but only in the CTRL group. This result could indicate that changes in the Krebs cycle correlated to the growth substrate supplemented with chestnut shells. Interestingly, in the previous manuscript, the protein and lipid contents of TRT1 were higher than in CTRL, suggesting fumarate consumption in metabolic pathways, such as amino acid or lipid synthesis [14]. To date, several articles in the literature have explored the metabolic processes of mealworms; however, only one study before ours employed NMR spectroscopy for this. Melis et al. [13] investigated the influences of dried brewers’ spent grains as an innovative substrate for wheat bran in the larvae’s metabolic response. It is noteworthy that both chestnut shells and dried brewers’ spent grains contain bioactive molecules that confer functional activity and can be valorized for animal nutrition in order to promote health [19]. In our study, larvae reared on chestnut shells showed a similar metabolic response observed by Melis et al., albeit with slight differences. It has been previously observed that dried brewers’ spent grains caused a decrease in fumarate levels in larvae compared to the control group.

Higher levels of acetate were found in the TRT groups compared to the CTRL. Acetate is a precursor of the carboxylic acids used to synthesize polyketides, which are recognized as important for insect defense [20]. In the previous article, TRT2 revealed a higher survival rate than the CTRL. Comparable profiles of other organic acids were found in larvae fed chestnut shells, with significant differences in larvae receiving only wheat bran, suggesting that the inclusion of chestnut shells might have influenced the metabolism of these compounds or led to selective absorption of dietary constituents.

Regarding sugars, a lower level of trehalose was found in TRT1 compared to CTRL, with numerical averages lower in TRT2 compared to the CTRL group. Glucose was detected in both chestnut shell groups, and it was absent in the CTRL. This is probably due to an increase in metabolic processes induced by the chestnut shell, leading to the transformation of trehalose into glucose. However, this specific mechanism should be further investigated [21]. Observed results of the sugar profile suggest the capacity of the chestnut shells to influence the energy balance of the insects.

Differences between TRT and CTRL groups were observed in nitrogen bases and their derivatives. The lower AMP content in the TRT groups indicates reduced metabolic activity in larvae grown on the innovative substrate. Uridine was exclusively detected in TRT1 and TRT2, which aligns with the observed decrease in UMP, UDP, and UTP levels compared to the CTRL group. The CTRL group, in contrast, did not show traces of uridine but registered significantly higher levels of UMP, UDP, UTP, and AMP, exceeding 201 mg/100 g of insect meals (on a DM basis). In insects, a decrease in ATP could result in reduced production of UTP from UDP phosphorylation, which leads to higher levels of uridine. Our results suggest that the larvae exhibit different energy demands based on the growth conditions provided by the innovative substrate.

The inclusion of chestnut shells did not alter the free fatty acid composition since comparable levels of all fatty acid groups were observed between TRT1 and CTRL samples. Significant differences were only detected in sterols content, whose value decreased in the TRT2 group. Further studies will be needed to investigate the complete lipid profile after chestnut shell supplementation in the growth substrate of *T. molitor* larvae.

Similar levels of compounds such as ethanolamine, choline, and phosphorylcholine were observed in TRT1 and TRT2, whereas significant differences were noted compared to the CTRL group. The observed reduction in phosphocholine levels, alongside the increase in choline in the TRT groups, indicate modifications in glycerophospholipid metabolism, suggesting a diet-dependent metabolic response. A previous study by Bridges (1972) [22] has shown that larvae needed choline to attain optimal growth during their juvenile stage.

Finally, PCA analysis showed separate clusters considering all the detected metabolites between larvae reared on chestnut shells and larvae fed with traditional substrate, with the most important discriminants being uracil, uridine, glucose, and fumarate. This result further confirms that chestnut shell supplementation in the growth substrate could affect the metabolism of *T. molitor*.

In line with the previous manuscript [14], which confirmed the role of the dietary sources in shaping the amino acid profile of insect meals, the present study showed the influence of chestnut shells on the other metabolic pathways of *T molitor*. The enrichment analysis showed that in the control and treatment groups, the metabolism of valine, leucine, and isoleucine was mainly influenced by the addition of chestnut shells in the growing substrates. Specifically, the concentration of alanine was significantly higher in TRT1 compared to CTRL, while leucine was lower in TRT2 compared to CTRL [14]. Additionally, glutamine levels were reduced in both TRT1 and TRT2, whereas pyroglutamate was present in the treatment groups but absent in the control group, indicating a modulation in the alanine, aspartate, and glutamate metabolic pathways [14].

According to our results, NMR spectroscopy provided detailed detection of a wide range of metabolites in insect meals reared on substrates supplemented with chestnut shells without altering the sample’s composition compared to other laboratory techniques. Therefore, NMR could be considered a valuable approach to investigate the metabolic profile and metabolic change response to dietary variations in insects. This study confirmed that the growth substrate could influence the performance and the nutritional and metabolomic characteristics of the *T. molitor* meal. The use of chestnut shells, rich in bioactive molecules such as polyphenols, is advantageous because being a waste product allows for cost reduction and a contemporary improvement in the functionality of insect meals. Other studies are necessary to further identify metabolomic markers that could potentially serve as detection methods to ensure the characteristics of the administered growth substrate in insect farming.

## 5. Conclusions

This study used NMR spectroscopy to explore how chestnut shell supplementation affected the metabolic profile of *Tenebrio molitor* larvae reared on innovative growth substrates. Key findings include the absence of fumarate and the presence of uracil, uridine, and glucose in TRT1 and TRT2 larvae meal, suggesting modifications in the energy metabolism without altering the lipidic profile by supplementing chestnut shell. This study supports the use of NMR spectroscopy for detailed metabolic profiling with a relatively simple sample preparation. Chestnut shells offer a cost-effective strategy to enhance the survival rate of larvae and improve the functional characteristics of the resulting meals by modulating insect metabolism. Further research should focus on identifying specific metabolomic markers to optimize insect farming practices.

## Figures and Tables

**Table 1:** Chemical composition of chestnut shell used for the experimental substrate formulation of TRT1 and TRT2.

Raw Ingredients	DM (%)	Ash (%)	CP (%)	EE (%)	CF (%)	NSCs (%)
Chestnut shell	93.39 ± 0.05	1.63 ± 0.02	3.72 ± 0.07	0.80 ± 0.03	17.09 ± 0.89	70.16 ± 0.83

All values are expressed as percentages of dry matter (DM) and expressed as mean ± standard deviation (*n* = 3). DM: Dry matter; Ash: Ashes; CP: Crude protein; EE: Ether extract; CF: Crude fiber; NSCs: Non-structural carbohydrates.

**Table 2:** Analytical composition of the experimental growth substrates administered to larvae (control: CTRL; treatment 1: TRT1; treatment 2: TRT2) during the experimental trial (from day 0 to day 14).

Experimental Group ^1^	DM (%)	Ash (%)	CP (%)	EE (%)	CF (%)	NSCs (%)
CTRL	90.62 ± 1.26	6.09 ± 1.86	17.00 ± 0.42 ^a^	2.93 ± 0.94	11.87 ± 4.30	62.11 ± 3.77
TRT1	91.89 ± 1.39	7.98 ± 1.65	14.48 ± 0.20 ^b^	1.65 ± 0.05	12.26 ± 0.97	63.62 ± 2.14
TRT2	91.63 ± 1.35	6.55 ± 0.63	15.09 ± 0.27 ^b^	1.73 ± 0.20	14.85 ± 0.43	61.78 ± 0.66
*p*-Value	0.5094	0.3393	0.0001	0.0523	0.3647	0.6572

^1^ CTRL: control group reared on wheat bran; TRT1: treatment group fed wheat bran supplemented with 12.5% *w*/*w* chestnut shell; TRT2: treatment group fed wheat bran supplemented with 25% *w*/*w* chestnut shell. All values are expressed as percentages of dry matter (DM) and expressed as mean ± standard deviation (*n* = 3). Means within a column with different lowercase letters are significantly different (ANOVA and Kruskal–Wallis tests, *p* < 0.05). DM: Dry matter; Ash: Ashes; CP: Crude protein; EE: Ether extract; CF: Crude fiber; NSCs: Non-structural carbohydrates.

**Table 3:** Metabolites and relative ^1^H NMR signals (chemical shift, multiplicity, *J*-coupling constant) selected for quantitative analysis in Bligh–Dyer hydroalcoholic and organic extracts.

Metabolite	^1^H NMR Signal (ppm), Multiplicity [*J*(Hz)]	Metabolite	^1^H NMR Signal (ppm), Multiplicity [*J*(Hz)]
Hydroalcoholic extract metabolites			
Lactate	1.34, d [6.6]	Trehalose	5.20, d [3.8]
Acetate	1.92, s	Fumarate	6.53, s
Citrate	2.56, d [15.9]	Uracil	5.80, d [7.6]
Ethanolamine	3.15, t [5.3]	Uridine	5.90, m
Choline	3.21, s	UMP/UDP/UTP	5.99, m
Phosphorylcholine	3.23, s	Adenosine	6.08, d [6.2]
Taurine	3.43, t [6.5]	AMP	6.14, d [6.0]
Glycerol	3.66, dd [11.7; 4.3]	Formate	8.46, s
Glucose	4.66, d [8.0]		
Organic extract metabolites			
Sterols	0.60–0.71	Di-unsaturated fatty acids	2.76, t [7.1]
Total Unsaturated fatty acids	1.92–2.12	Tri-unsaturated fatty acids	2.80, t [6.9]
Total fatty acids	2.21–2.42		

## Data Availability

The original contributions presented in the study are included in the article, further inquiries can be directed to the corresponding author.
